# Prognostic significance of nephrectomy in metastatic renal cell carcinoma treated with systemic cytokine or targeted therapy: A 16-year retrospective analysis

**DOI:** 10.1038/s41598-018-20822-2

**Published:** 2018-02-14

**Authors:** Sung Han Kim, Kyung-Chae Jeong, Jae Young Joung, Ho Kyung Seo, Kang Hyun Lee, Jinsoo Chung

**Affiliations:** 1 Department of Urology, Center for Prostate Cancer, Research Institute and Hospital of National Cancer Center, Goyang, Korea; 2 Biomolecular Function Research Branch, Research Institute, National Cancer Center, Goyang, Gyeonggi-do Korea

## Abstract

We compared progression-free survival (PFS) and overall survival (OS) among 292 metastatic renal cell carcinoma (mRCC) patients either undergoing nephrectomy (Nx, 61.6%) or not (non-Nx, 38.4%), stratified according to the MSKCC and Heng risk models, treated with either immunotherapy (IT, 45.2%) or targeted therapy (TT, 54.8%) between 2000 and 2015. During the follow-up duration of 16.6 months, PFS/OS of the Nx (6.0/30 months) and non-Nx (3.0/6.0 months) groups were significantly different despite differences among baseline parameters (p < 0.05). The intermediate- and poor-risk patients defined using either model showed significantly longer PFS and OS in the Nx group than in the non-Nx group (p < 0.05). After stratifying groups by systemic therapy and risk models, both the Nx and non-Nx groups showed no significant differences in intermediate and poor-risk models (p > 0.05). In both synchronous and metachronous mRCC patients, both PFS and OS showed similar survivals; the Nx group had significantly longer PFS and OS than the non-Nx group, even after considering each systemic therapy and prognostic model. Nx showed a significant positive benefit in PFS and OS compared to no Nx upon patient stratification according to the MSKCC and Heng risk models. The metastatic type did not significantly affect survival between the two groups.

## Introduction

The standard therapy for metastatic renal cell carcinoma (mRCC) has been systemic therapy including cytokine immunotherapy (IT) in the past decade and systemic targeted therapy (TT) in recent decades. However, the outcomes of systemic therapy are unsatisfactory with a dismal 5-year overall survival (OS) rate of <20%, because RCC is resistant to radiotherapy, chemotherapy, and immunotherapy^1^. Complete surgical resection of the tumor (radical or partial) is the only known curative therapy for RCC localized within the kidney^2^.

Regarding mRCC, cytoreductive nephrectomy (CNx) is often indicated as part of an integrated therapeutic management strategy for mRCC to reduce the potential tumor burden as well as control cancer-related symptoms, including hemorrhage, pain, and paraneoplastic syndrome^3^. CNx has also been proven to improve prognosis of mRCC treated either with IT or TT; however, randomized clinical trials have not yet clarified the definite survival benefit of CNx with TT. Several large-scaled retrospective studies and meta-analyses have shown the favorable effects of CNx on survival with spontaneous regression of metastasis in up to 4% of cases^4–7^, a decreased risk of death and a 5.8-month survival advantage before IT^8–10^.

In this retrospective study, we aimed to evaluate the prognostic significance of Nx on progression-free survival (PFS) and OS in patients with mRCC through stratification by types of systemic therapy, metastatic types, and the widely used prognostic risk models (the Memorial Sloan-Kettering Cancer Center [MSKCC] and the International Metastatic Renal Cell Carcinoma Database Consortium [IDMC, also known as Heng]^11^ risk models).

## Materials and Methods

### Ethics statement

The Institutional Review Board of the National Cancer Center (IRB No. NCC 2015-0212) approved this retrospective study and waived the need for written consent. All patient data were anonymized and de-identified prior to analysis. All study protocols were performed in accordance with the tenets of the ethical guidelines and regulations of the ‘World Medical Association Declaration of Helsinki-Ethical Principles for Medical Research Involving Human Subjects.’

### Criteria for patients

We analyzed the data of 292 mRCC patients treated with first-line systemic therapy using either TT (N = 160, 54.8%) or IT (N = 132, 45.2%) including sunitinib, sorafenib, pazopanib, temsirolimus as first-line TT and interferon-alpha and interleukin-2 as IT between 2000 and 2015, as cited in previously published reports^12^. A total of 183 (61.6%) patients who underwent Nx and 109 (38.9%) patients who did not (non-Nx) were enrolled for the evaluation PFS and OS, and were stratified by their MSKCC and Heng (favorable, intermediate, and poor) risk criteria and metastatic types (synchronous and metachronous type), as well as types of systemic therapies used (IT or TT). Furthermore, patient records were retrospectively extracted from the data prospectively recorded by our institutional RCC database to obtain baseline information regarding age, body mass index, ECOG–performance status (ECOG-PS), TNM stage, cell histology, Fuhrman nuclear grade, and survival outcomes. The radical nephrectomy (RNx) or CNx procedure was performed by open access or laparoscopically by a single surgeon (J.C.). If CNx was not performed, histological confirmation of RCC was performed by biopsies from primary or metastatic lesions or both. The RECIST criteria 1.1^13^ were used for the evaluation of response to systemic therapies after each therapeutic regimen and follow-up protocol cited in previous reports^11^.

### Statistical analysis

The baseline characteristics are presented as frequency (percentage) for categorical variables and median (range) for continuous variables. Differences in distributions were compared between SM and MM using the Student’s t-test, Pearson’s Chi-square test, Fisher’s exact test, and Log-rank test as appropriate. Survival curves and their differences are presented using the Kaplan-Meier curve and log-rank test to analyze times to progression (PFS) for first-line systemic therapy and death (OS) after systemic therapy according to the number of MSKCC and Heng risk factors. All results were considered statistically significant when two-sided p-values were <0.05 using SAS 9.2 software (SAS Institute Inc., Cary, NC, USA).

### Data availability

The datasets generated and/or analyzed during the current study are available from the corresponding author upon reasonable request.

## Results

During a median 10.0- months of treatment and 16.6 months of follow-up, the median PFS/OS was 11.0 (8.0-14.0)/4.0 (2.8–5.2) months with an objective response rate of 17.5% and disease control rate of 63.4% after first-line therapy (Table 1). The median number of metastatic organs was 2.0 (range, 1–5) with the lung being the most frequently metastasized organ (75.7%), followed by bones (45.2%), lymph nodes (24.3%), brain (18.8%), and the liver (17.1%). A total of 106 patients had only one metastatic site (36.2%) and 98 (33.3%) patients had at least two or more metastatic organs. The overall patients’ Charlson comorbidity index was a median of 7.0 (range, 6–13) with hypertension (43.8%) and diabetes (22.3%) being the most frequent underlying diseases among enrolled patients.

**Table 1:** Baseline demographics (N = 292).

Parameter	N or median	Percentage or range
Age (years)	58.0	20–82
Sex (Male/Female)	234/58	80.1/19.9
Metastatic type, SM/MM	171/121	58.6/41.4
ECOG, 0–1/2–3/unknown	239/18/29	83.9/6.2/9.9
Karnofsky Performance Score		
≥80 vs. 50–70 vs unknown	145/21/126	49.7/7.1/43.2
Charlson comorbidity index	7.0	6–13
Heart disease	18	6.2
Hypertension	128	43.8
Cerebrovascular disease	5	1.7
Peripheral vascular disease	2	0.7
Chronic pulmonary disease	5	1.7
Diabetes	65	22.3
Liver disease	12	4.1
Renal disease	3	1.7
Depression	2	0.7
Other previous cancer history	8	2.7
Metastatic sites		
Lung	221	75.7
Liver	50	17.1
Lymph nodes	71	24.3
Bones	132	45.2
Brain	55	18.8
Other sites	73	25.0
Number of metastatic organs	2.0	1–5
1	106	36.2
2	98	33.6
3	58	19.9
4	24	8.2
5	6	2.1
MSKCC risk		
Favor/Inter/Poor/Unknown	42/197/49/4	14.4/67.7/16.8/1.4
Heng risk		
Favor/Inter/Poor/Unknown	40/203/45/4	13.7/69.5/15.4/1.4
Treatment duration (mo.)	10.0	1–149
Nephrectomy	183	62.7
T stage/		
Clinical T1-T2/T3–4/Tx	82/53/157	28.1/18.2/53.7
Pathologic T1-T2/T3–4/Tx	50/45/88	27.3/24.6/48.1
N stage		
Clinical N0/N1/Nx	69/43/180	23.7/14.7/61.6
Pathologic N0/N1/Nx	53/7/123	29.0/3.8/67.2
Fuhrman grade		
Clinical, Gr.1–2/3–4/unknown	5/54/233	1.7/18.5/79.8
Pathologic, Gr.1–2/3–4/unknown	34/61/108	18.6/33.3/59.0
Primary Tx drug		
Targeted therapy (TT)	160	54.8
Immunotherapy (IT)	132	45.2
(IT first, followed by TT)	(34)	(11.6)
Treatment-free interval <1 yr	158	54.1
Follow-up duration (months)	16.6	1–167.5
First-line RECIST criteria		
Complete response	6	2.1
Partial response	45	15.4
Stable disease	134	45.9
Progressive disease	107	36.6
Survival/Death	45/247	15.5/84.5

The median PFS/OS for IT and TT were 2.0 (1.1–2.9) months/10.0 (6.8–13.2) months and 5.0 (3.5–6.5) months/13.0 (8.4–17.6) months, respectively (OS for IT-TT, 24.0 [11.2–36.8] months). The Nx group had significantly longer PFS/OS (6.0/30.0 months) than the non-Nx group (3.0/8.0 months, p < 0.001, Supplementary Figure 1). The comparison of PFS/OS between IT and TT according to the prognostic model showed significant differences. TT had better prognostic effects on both PFS (9.0 and 4.0 months) and OS (32.0 and 9.0 months) than IT (PFS, 4.0 and 2.0 months; OS, 26.0 and 6.0 months) among intermediate and poor risks, and the IT-TT group had the best PFS/OS among all MSKCC and Heng risk groups (p < 0.05, Table 2 and Supplementary Figure 2). Despite the significant differences in baseline characteristics between the Nx and non-Nx groups, including those in metastatic type, ECOG-performance status, number of MSKCC and Heng risk factors, treatment duration, clinical and pathologic T stage, first line systemic therapy, rate of treatment-free interval <1 year, and survival rate (p < 0.05, Table 3), patients in the Nx group had significantly prolonged PFS/OS compared to the non-Nx group (p < 0.05, Table 4).

**Table 2:** PFS and OS according to first-line systemic treatments including sequential treatment, and prognostic risk models.

Risk group	1^st^-line treatment	n	Median (mos)	95% CI	p-value
MSKCC risk group					
PFS according to systemic therapy and MSKCC risk group					
Favorable	IT	19	7	2.9–11.1	<0.001
	TT	22	11	4.6–17.4	
Intermediate	IT	84	4	3.0–5.0	
	TT	113	6	3.9–8.1	
Poor	IT	27	2	1.3–2.7	
	TT	22	3	1.4–4.6	
OS according to systemic therapy and MSKCC risk group					
Favorable	IT	11	49.0	5.8–92.2	<0.001
	IT → TT	9	82.0	34.9–129.1	
	TT	22	51.0	29.4–72.6	
Intermediate	IT	63	17.0	9.4–24.6	
	IT → TT	21	28.0	11.7–44.3	
	TT	111	18.0	13.9–22.1	
Poor	IT	23	6.0	4.9–7.1	
	IT → TT	4	6.0	1–34.4	
	TT	21	5.0	2.8–7.2	
Heng risk group					
PFS according to systemic therapy and Heng risk group					
Favorable	IT	19	7	3.0–11.0	<0.001
	TT	20	11	1.0–21.7	
Intermediate	IT	95	4	2.9–5.1	
	TT	108	8	5.3–10.7	
Poor	IT	16	2	1.1–2.9	
	TT	29	3	1.8–4.2	
OS according to systemic therapy and Heng risk group					
Favorable	IT	12	49	18.5–79.6	<0.001
	IT → TT	8	83	80.9–85.1	
	TT	20	53	24.0–82.0	
Intermediate	IT	70	14	8.2–19.8	
	IT → TT	23	28	20.2–35.8	
	TT	106	20	13.3–26.7	
Poor	IT	13	6	3.7–8.3	
	IT → TT	3	10	3.6–16.4	
	TT	28	5	3.7–6.3	

TT, targeted therapy; IT, immunotherapy.

**Table 3:** Comparison of baseline characteristics between nephrectomy and non-nephrectomy groups (N = 292).

Parameter	Nx (n = 183)	Non-Nx (n = 109)	p-value
Age (years)	55.9 ± 10.6	55.6 ± 12.2	0.057
Sex (Male/Female)	147/36 (80.3/19.7)	87/22 (79.8/20.2)	1.000
Metastatic type, SM/MM	78/105	109/0	<0.001
ECOG,-PS 0–1/2–3/unknown	146/12/23 (90.6/6.7/12.7)	99/6/4 (90.8/5.5/3.7)	0.015
Karnofsky-PS ≥80/50–70/unknown	153/5/5 (83.6/8.2/8.2)	98/6/5 (89.9/5.5/4.6)	0.231
MSKCC risk	40/133/8/2	2/64/41/2	<0.001
Favor/Inter/Poor/Unknown	(22.1/73.5/4.4/1.0)	(1.8/58.8/37.6/1.8)	
Heng risk	38/134/9/2	2/69/36/2	<0.001
Favor/Inter/Poor/Unknown	(20.8/73.3/4.9/1.0)	(1.8/63.4/33.0/1.8)	
Treatment duration (mo.)	29.2 ± 11.7	27.9 ± 9.6	<0.001
Clinical T stage			0.001
T1-T2/T3-T4/Tx	43/14/126(23.5/7.6/68.9)	39/39/31(35.8/35.8/28.4)	
Clinical N stage			0.258
N0/N1/Nx	30/12/141(16.4/6.6/77.0)	39/31/39(35.8/28.4/35.8)	
Primary Tx drug			0.034
TT/IT/IT-TT	90/67/26(49.2/36.6/14.2)	70/31/8(64.2/28.4/7.3)	
Treatment-free interval <1 yr	58 (34.3)	100 (97.1)	<0.001
First-line RECIST criteria			0.103
CR/PR/SD/PD/Unknown	33/6/29/86/26 (18.3/3.3/16.1/47.8/14.4)	16/0/16/48/26 (15.1/0/15.1/45.3/24.5)	
Survival/Death	35/148 (19.1/80.9)	10/98 (9.3/90.7)	0.029

**Table 4:** PFS and OS according to nephrectomy status and prognostic risk group.

Risk group	Nephrectomy status	n	Median (mos)	95% CI	p-value
PFS according to nephrectomy status and MSKCC risk group					
Favorable	Nephrectomized	41	8	5.3–10.7	NA
Intermediate	Nephrectomized	133	5	4.0–6.0	0.012
	non-nephrectomized	64	4	2.1–5.9	
Poor	Nephrectomized	8	11	6.4–15.6	
	non-nephrectomized	41	2	1.4–2.	
OS according to nephrectomy status and MSKCC risk group					
Favorable	Nephrectomized	41	51.0	12.0–90.0	NA
Intermediate	Nephrectomized	132	25.0	18.2–31.8	<0.001
	non-nephrectomized	63	10.0	8.1–11.9	
Poor	Nephrectomized	8	20.0	8.9–40.8	
	non-nephrectomized	40	5.0	3.8–6.4	
PFS according to nephrectomy status and Heng risk group					
Favorable	Nephrectomized	41	9	6.0–12.1	NA
Intermediate	Nephrectomized	69	5	3.9–6.1	0.008
	non-nephrectomized	134	3	1.4–4.6	
Poor	Nephrectomized	9	4	1.0–7.8	
	non-nephrectomized	36	2	1.4–2.6	
OS according to nephrectomy status and Heng risk group					
Favorable	Nephrectomized	41	59.0	21.4–96.6	NA
Intermediate	Nephrectomized	133	27.0	20–34	<0.001
	non-nephrectomized	68	9.0	7–11	
Poor	Nephrectomized	9	20.0	4.8–35.2	
	non-nephrectomized	35	5.0	3.8–6.2	

NA, comparison between the nephrectomized and non-nephrectomized groups not available.

After stratification of prognostic risks and systemic therapies, patients in the Nx and non-Nx groups had insignificantly different PFS and OS, especially among the poor-risk patients with no benefits in PFS and OS (p > 0.05, Table 5). Regarding the comparison between Nx and non-Nx for SM and MM, the Nx group had significantly better PFS (5.0 vs. 3.0 months) and OS (22.0 vs. 7.0 months) than the non-Nx group regardless of the types of systemic therapy and prognostic risk models (p < 0.05, Supplementary Tables 1 and 2).

**Table 5:** PFS and OS according to Nx status, type of systemic treatment and prognostic risk models

Risk group	1^st^-line	Nephrectom	n	Median	95% CI	p-value
PFS according to nephrectomy status and MSKCC risk group						
Favorable	IT	Nx	14	16	6.3–25.7	NA
	TT	Nx	27	7	2.4–11.6	
Intermediate	IT	Nx	70	4	3.1–4.9	0.159
		non-Nx	18	5	1.0–7.6	
	TT	Nx	86	6	4.8–7.2	
		non-Nx	18	4	1.0–12.8	
Poor	IT	Nx	12	2	1.0–3.1	
		non-Nx	14	2	2.1–11.9	
	TT	Nx	10	7	1.0–3.0	
		non-Nx	12	2	1.0–3.2	
OS according to nephrectomy status and MSKCC risk group						
Favorable	IT	Nx	10	49	25.6–72.4	NA
	IT → TT	Nx	4	82	1.0–168.9	
	TT	Nx	28	51	22.0–80.0	
Intermediate	IT	Nx	51	22	13.5–30.5	0.956
		non-Nx	14	11	4.9–17.1	
	IT → TT	Nx	19	7	11.8–32.2	
		non-Nx	4	10	1.0–13.1	
	TT	Nx	84	22	7.3–26.7	
		non-Nx	18	9	4.9–13.1	
Poor	IT	Nx	7	4	1.4–6.6	
		non-Nx	13	6	4.7–7.3	
	IT → TT	Nx	2	5	5.0–5.0	
		non-Nx	3	5	4.2–7.8	
	TT	Nx	7	9	6.4–11.6	
		non-Nx	15	8	3.0–5.6	
PFS according to nephrectomy status and Heng risk group						
Favorable	IT	Nx	16	16	4.6–27.4	NA
	TT	Nx	23	6.0	2.7–9.3	
Intermediate	IT	Nx	68	4	3.1–4.9	0.080
		non-Nx	24	2	1.0–4.0	
	TT	Nx	90	6	4.4–7.6	
		non-Nx	16	6	1.5–10.5	
Poor	IT	Nx	12	2	1.0–3.6	
		non-Nx	8	2	1.0–3.8	
	TT	Nx	10	3	1.1–4.9	
		non-Nx	14	2	1.3–2.7	
OS according to nephrectomy status and Heng risk group						
Favorable	IT	Nx	11	49	11.0–101.7	NA
	IT → TT	Nx	5	82	32.6–131.4	
	TT	Nx	24	53	9.5–96.5	0.898
Intermediate	IT	Nx	51	22	13.5–30.5	
		non-Nx	20	14	5.3–8.8	
	IT → TT	Nx	16	15	7.7–22.3	
		non-Nx	5	11	1.0–20.7	
	TT	Nx	86	28	20.1–35.9	
		non-Nx	18	19	5.9–12.1	
Poor	IT	Nx	6	5	1.4–8.6	
		non-Nx	7	6	4.9–7.1	
	IT → TT	Nx	4	5	5.1–16.5	
		non-Nx	2	2	1.0–6.4	
	TT	Nx	9	9	1.0–17.8	
		non-Nx	15	6	2.3–9.7	

NA, comparison between the nephrectomized and non-nephrectomized groups not available.

## Discussion

The therapeutic concepts of Nx in metachronous and synchronous mRCC are different, although radical (or partial) Nx for localized RCC and CNx for mRCC both have a significant survival gain^6,14,15^. Radical (or partial) Nx removes all cancer cells en-bloc within the primary kidney rendering the patient completely free of cancer until metastasis or recurrence occurs. Recurrent or metastasized cancers in metachronous mRCC are composed of dormant cancer cells that have re-appeared or new cancer cells with different metabolic activity in metastatic lesions, and create a tumor microenvironment that is different from lesions in localized RCC. Meanwhile, CNx for synchronous mRCC removes the primary loco-regional tumor as much as possible to decrease the tumor burden in order to achieve better responses to systemic therapy and focuses more on the metastatic lesions^16,17^. Although CNx does not guarantee a surgically curable status, a few selected patients achieved complete remission after CNx in combination with targeted therapy^18^.

The effects of Nx for mRCC with regard to the prognostic risks, metastatic types, and type of systemic therapy were compared to those of no Nx. Nx had significant benefits for OS compared to no Nx in intermediate risk patients with mRCC (Table 4) despite the more favorable baseline characteristics of patients in the Nx group (p < 0.05, Tables 1 and 2). The Nx group was mostly consisted of patients with favorable risk group and metachronous type of metastasis. However, the Nx and no Nx groups failed to show any significant differences in PFS among any risk groups and in OS within the poor-risk group (Table 5).

Additional findings on the metastatic types showed that they did not significantly affect the prognostic survival differences between patients who underwent Nx and those who did not (Supplementary Tables 1 and 2). The synchronous and metachronous sets showed almost no difference in the survival of patients who underwent Nx and those who did not. Both sets of patients with mRCC had a significantly better PFS and OS after Nx than without Nx upon stratification by types of systemic therapy and in the prognostic risk models. This implied that Nx was beneficial for both synchronous and metachronous mRCCs when the patient’s surgical condition and disease status allowed it, and thus, was a significant factor for better survival in mRCC. Regarding the type of systemic therapy, in both metastatic types, Nx with IT or with TT resulted in similar median OS of 20 months and 22–25 months, respectively, and no Nx with IT or TT resulted in similar OS (6 months vs. 9 months) within the MSKCC and Heng risk models (Supplementary Tables 1 and 2).

The recent development of targeted agents using a genetic analysis technique permitted prolonged survival of mRCC patients compared to that with IT, especially in the intermediate Heng risk and Nx groups with more than two-fold increase in OS, (p < 0.001, Table 2), which is similar to the results of this study. Moreover, Nx with adequate and proper selection of the targeted therapeutic agents as well as of patients with a prognostic model would provide the best opportunities for survival compared to no Nx^6,15^. Bamias *et al*.^19^ showed that patients with primary mRCC treated with sunitinib and CN had a significantly longer OS than patients who did not undergo CNx (23.9 vs. 9 months), and found the Heng and MSKCC risk models to be associated with prognostic significance. The present study also demonstrated that in favorable and intermediate risk patients with general conditions susceptible to Nx, TT had much greater chances of controlling tumors in mRCC patients than non-Nx. Other retrospective studies^20–22^ including a Surveillance, Epidemiology, and End Results (SEER) database study^21^ have suggested CNx as an independent factor of OS with 19 months of improvement after CNx in the TT era even after adjusting for established prognostic risk factors (vs. 13 months in the IT era)^20^. Finally, two famous randomized phase 3 trials namely, CARMENA with Nx followed by sunitinib vs. sunitinib alone and SURTIME, with immediate vs. deferred (after 3 complete cycles of sunitinib) CNx^23^ are currently ongoing and would provide definite answers to questions on the benefit and timing of CNx for mRCC treated with TT^24^.

Discussions on CNx have included the indications for CNx by properly selecting patients. Regarding the better prognosis of CNx, the current indications for mRCC were patients with good performance status, large primary tumors accounting for most of the disease volume, and low metastatic volume, but not patients with poor PS or Heng or MSKCC poor-risk disease, with relatively small primary tumors and high metastatic volume, and/or with sarcomatoid tumors^4,23^. There is little doubt that patients with solitary tumors or oligometastasis could either potentially be cured by CNx and complete resection of metastatic sites, or benefit from a substantial delay and reduction in associated side-effects of systemic therapy before further progression requires medication^6,16–18^.

However, because of the clinical and biological heterogeneity of synchronous mRCCs and the lack of biomarkers, not all patients with mRCC benefited from CNx, especially among the poor-risk group^25^. CNx in patients who have a poor performance (ECOG 2/3 patients) might serve a palliative function, but it should be performed with caution because of the poor outcomes in such patients^26^. This study did not show a significant beneficial effect on survival compared to that in the non-CNx poor-risk group, similar to the studies by Choueiri and Heng^11,21^. Heng *et al*. also found that most patients with 4 or more IMDC risk factors did not benefit from tumor removal^22^.

Despite the CNx effect on survival, the use of CNx declined to 34–38% in 2005–2010 since the introduction of TT, suggesting that more patients would eventually be treated with the primary tumor *in situ*^11,27^. The reason for this decline was that, unlike IT, TT alone could control the disease, particularly at the primary tumor site; thus, Nx might not be necessary. This might avoid the morbidity and mortality associated with surgery followed by a postoperative delay in starting systemic treatment allowing further disease progression.

Another issue regarding CNx was its effect on mRCC with the combination of TT in a neoadjuvant (in the meaning of “presurgical”) setting with delayed CNx. The theoretical advantage of neoadjuvant TT with delayed CNx was downsizing the primary renal tumor and rapid initiation of effective systemic therapy without any delay associated with planning, performing, and recovering from Nx after supportive care for improving general conditions^143^. Moreover, patients with primary refractory disease would be accurately identified, and therefore, might have an opportunity to promptly switch to another targeted agent. However, it should be noted that approximately 30% of patients failed to go on the planned Nx in neoadjuvant trials due to changes in the performance state of individuals and downsizing of the tumor with TT seemed to be quite modest (2–6% by the RECIST criteria), which was not enough to facilitate CNx^14^. The frequent occurrence of disease progression (approximately 33–37% by the RECIST criteria)^28^ during a surgery-related break in treatment was another important issue that occurred approximately 3–4 weeks postoperatively^29^. However, this neoadjuvant TT and delayed CNx might be one option for poor-risk patients with an ECOG score of 2 to 3 in the poor-risk group. TT might be proposed as the first-line treatment to these patients, especially with MSKCC intermediate-risk disease in a phase 2 trial with pazopanib; CNx should be considered only after an objective response to the systemic treatment^30^.

In addition, the rapid progression after cessation of TT with CNx remains unclear, as to whether it was due to the withdrawal of anti-angiogenic therapy before surgery, the release of growth factors after surgery, a combination of both, or if it was progression due to TT resistance. Translational research on primary tumor tissue has shown that withdrawal of sunitinib led to rapid endothelial cell proliferation^31^. This seems to support the hypothesis that a treatment break might trigger progression in some patients, although that patient population cannot be identified^4^. A tumor flare phenomenon suggested by Escudier *et al*.^32^ showed acceleration of tumor growth rate and induction of tumor flares after cessation of systemic treatment, which could negatively affect mRCC prognosis.

The limitations of this study were its retrospective design with a small number of poor-risk patients. Additionally, the intraoperative measures and further analyses of systemic agents, including pathological and metastatic information have not been accounted in the survival rate of Nx group. However, this is an evidence-based retrospective study providing the positive aspects of CNx in the TT era. A carefully selected group of patients with synchronous mRCC can obtain a clinical benefit with survival gain after combinational local surgery such as metastatectomy or radiation therapy with systemic TT.

## Conclusion

The prognostic significance of Nx for PFS and OS according to the MSKCC and Heng criteria showed a positive effect in the intermediate- and poor-risk groups among mRCC patients treated with first-line systemic therapies. A stratified comparison of PFS and OS by systemic therapies and metastatic types between the Nx and non-Nx groups showed no significant differences among those with poor risk; however, significant differences among those with intermediate risk were observed.

## Electronic supplementary material

Supplementary information
