# Supplementary material for: Prognostic significance of nephrectomy in metastatic renal cell carcinoma treated with systemic cytokine or targeted therapy: A 16-year retrospective analysis
Source: Sci Rep. 2018 Feb 14;8:2974. doi: 10.1038/s41598-018-20822-2 (PMC5813006; doi:10.1038/s41598-018-20822-2)
Supplement: Supplementary file 1 — Supplementary information [file 41598_2018_20822_MOESM1_ESM.pdf]

Prognostic significance of nephrectomy in metastatic renal cell carcinoma treated with systemic cytokine or targeted therapy: A 16-year retrospective analysis

Sung Han Kim[1+], Kyung-Chae Jeong[2+], Jae Young Joung[1], Ho Kyung Seo[1], Kang Hyun Lee[1], Jinsoo Chung[1]*

[1]Department of Urology, Center for Prostate Cancer, Research Institute and Hospital of National Cancer Center, Goyang, Korea

[2]Biomolecular Function Research Branch, Research Institute, National Cancer Center, Goyang, Gyeonggi-do, Korea

Supplementary table 1. Comparison of PFS and OS in SM group between with cytoreductive nephrectomy or non-cytoreductive nephrectomy according to nphrectomy status, first-line treatments and prognostic risk model

		PFS	95%CI	p-value	OS	95%CI	p-value
Nx		5	3.7-6.3	0.028	22	17.3-	<0.001
Non-Nx		3	2.2-3.8		7	5.2-8.8	
Treatment				0.002			<0.001
IT	Nx	4	1.6-6.4		20	12.2-	
	Non-Nx	2	1.4-2.6		6	4.2-7.8	
TT	Nx	9	5.4-12.6		25	13.4-	
	Non-Nx	4	2.2-5.8		9	7.0-11.0	
IT-TT	Nx	NA	NA		24	10.4-	
	Non-Nx	NA	NA		10	1.0-20.3	
MSKCC				0.047			0.024
Intermediate	Nx	5	3.8-6.2		22	16.1-	
	Non-Nx	4	2.1-5.9		10	8.1-11.9	
Poor	Nx	11	1.6-20.4		21	18.4-	
	Non-Nx	2	1.5-2.5		5	3.6-6.4	
Heng				0.028			0.035
Intermediate	Nx	6	4.87.2		22	16.0-27	
	Non-Nx	3	1.4-4.6		9	7-11	
Poor	Nx	3	1.0-7.1		20	7.5-46	
	Non-Nx	2	1.4-2.6		5	3.8-6.2	

NA, not available

Supplementary table 2. Comparison of PFS and OS in MM group between with radical nephrectomy or non-radical nephrectomy according to nephrectomy status, first-line treatments and prognostic risk model

		PFS	95%CI	p-value	OS	95%CI	p-value
Nx		22	17.3-26.7	<0.001	22	17.3-26.7	<0.001
Non-Nx		7	5.3-8.8		7	5.2-8.8	
Treatment				<0.001			<0.001
IT	Nx	20	13.8-26.2		20	12.2-27.8	
	Non-Nx	6	4.4-7.6		6	4.2-7.8	
TT	Nx	25	13.4-36.6		25	13.4-36.6	
	Non-Nx	9	7.0-11.0		9	7.0-11.0	
IT-TT	Nx	NA	NA		24	10.4-37.6	
	Non-Nx	NA	NA		10	6.7-20.3	
MSKCC				<0.001			<0.001
Intermediate	Nx	22	16.1-27.9		22	16.1-27.9	
	Non-Nx	10	8.1-11.9		11	8.1-11.9	
Poor	Nx	21	18.4-23.6		21	18.4-23.6	
	Non-Nx	5	3.6-6.4		5	3.6-6.4	
Heng				<0.001			<0.001
Intermediate	Nx	22	16.0-28.0		22	16.0-28.0	
	Non-Nx	9	7.0-11.0		9	7.0-11.0	
Poor	Nx	20	7.5-46.7		20	7.5-46.7	
	Non-Nx	5	3.8-6.2		5	3.8-6.2	

NA, not available

Supplementary figure 1. Comparison of (A) progression-free survival and (B) overall survival curves between nephrectomy and non-nephrectomy

A.

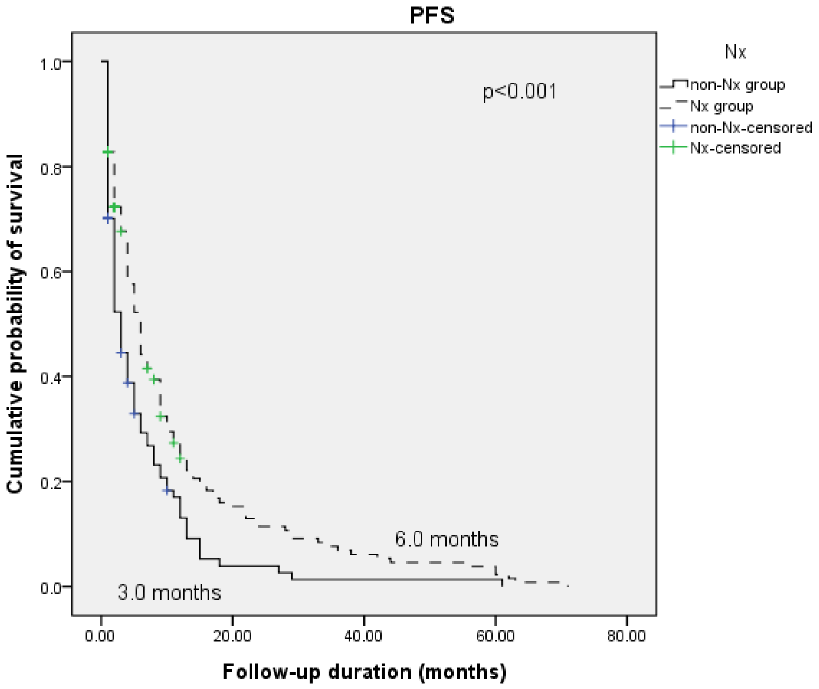

B.

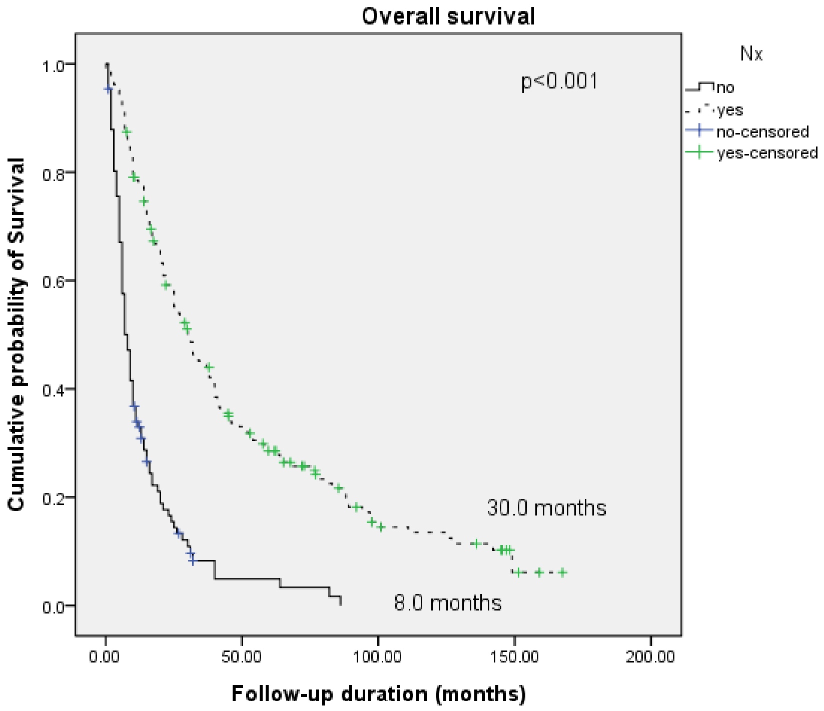

Supplementary figure 2. Comparison of (A, C) progression-free survival and (B, D) overall survival curves between nephrectomy and non-nephrectomy in immnuotherpy and of targeted therapy groups

A.

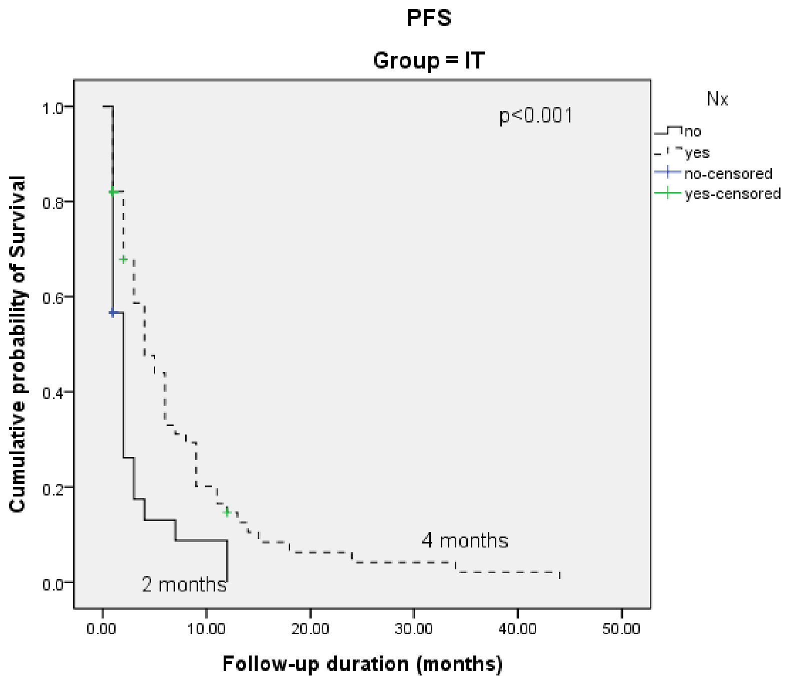

B.

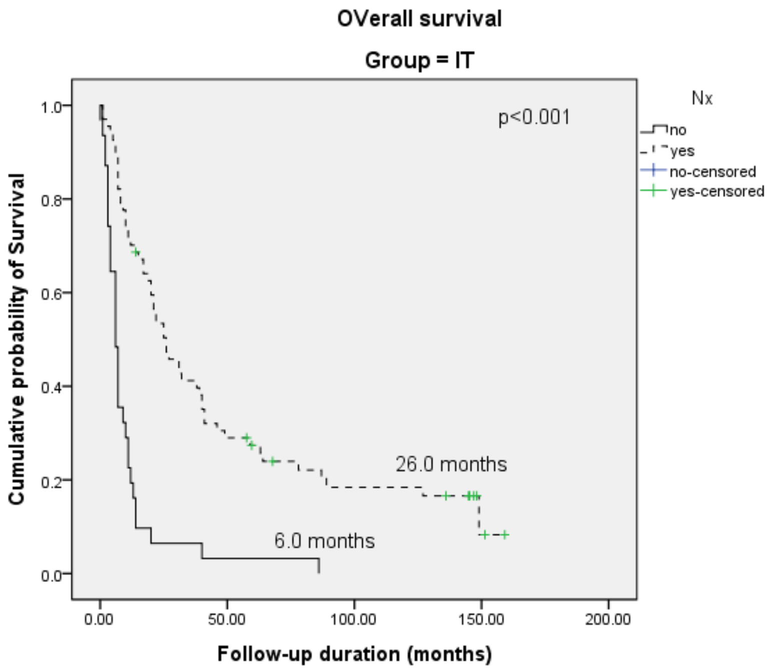

C.

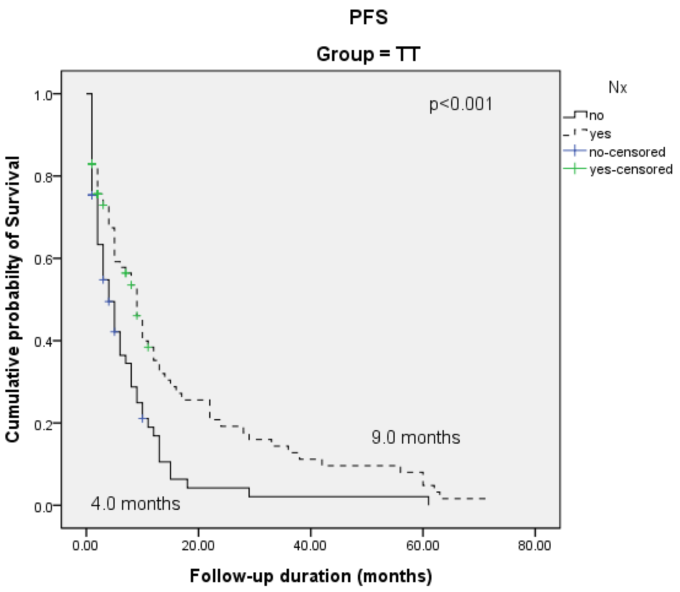

PFS
Group = TT
p<0.001
Nx
no
yes
no-censored
yes-censored
9.0 months
4.0 months
Cumulative probabilty of Survival
1.0
0.8
0.6
0.4
0.2
0.0
0.00
20.00
40.00
60.00
80.00
Follow-up duration (months)

D.

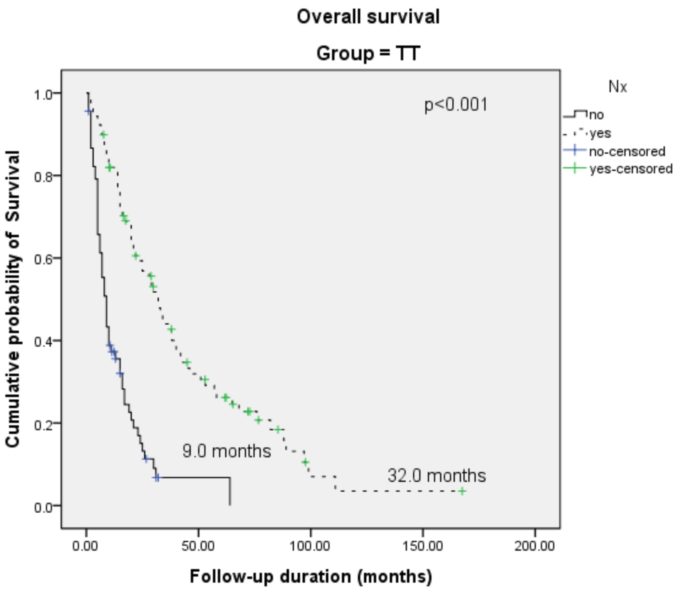

Overall survival
Group = TT
p<0.001
Nx
no
yes
no-censored
yes-censored
9.0 months
32.0 months
Cumulative probability of Survival
1.0
0.8
0.6
0.4
0.2
0.0
0.00
50.00
100.00
150.00
200.00
Follow-up duration (months)
